# Impact of P2X_7_ Purinoceptors on Goblet Cell Function: Implications for Dry Eye

**DOI:** 10.3390/ijms22136935

**Published:** 2021-06-28

**Authors:** Donald G. Puro

**Affiliations:** Departments of Ophthalmology & Visual Sciences and Molecular & Integrative Physiology, University of Michigan, Ann Arbor, MI 48105, USA; dgpuro@umich.edu

**Keywords:** P2X_7_ receptor/channels, exocytosis, membrane capacitance, conjunctival goblet cells

## Abstract

By providing ~70% of the eye’s refractive power, the preocular tear film is essential for optimal vision. However, its integrity is often jeopardized by environmental and pathologic conditions that accelerate evaporation and cause sight-impairing dry eye. A key adaptive response to evaporation-induced tear film hyperosmolarity is the reflex-triggered release of tear-stabilizing mucin from conjunctival goblet cells. Here, we review progress in elucidating the roles of ion channels in mediating this important exocytotic response. Much is now known about the modulatory impact of ATP-sensitive potassium channels, nonspecific cation channels and voltage-gated calcium channels. Recently, we discovered that during unremitting extracellular hyperosmolarity, P2X_7_ receptor/channels also become activated and markedly impair goblet cell viability. However, our understanding of possible adaptive benefits of this P2X_7_ activation remains limited. In the present study, we utilized high-temporal resolution membrane capacitance measurements to monitor the exocytotic activity of single goblet cells located in freshly excised rat conjunctiva. We now report that activation of P2X_7_ purinoceptors boosts neural-evoked exocytosis and accelerates replenishment of mucin-filled granules after exocytotic depletion. Thus, P2X_7_ activation exerts a yin-yang effect on conjunctival goblet cells: the high-gain benefit of enhancing the supply of tear-stabilizing mucin is implemented at the high-risk of endangering goblet cell survival.

## 1. Introduction

Visual perception begins at the preocular tear film where this interface between the external environment and the ocular surface plays a key role in focusing incoming light [1]. To effectively perform this important optical function, stability of the tear film is essential. However, the integrity of this complex fluid layer is threatened by a multitude of processes that accelerate its evaporation. Common environmental conditions such as the rapid movement of air and low ambient humidity cause transient preocular dryness. More persistent instability of the tear film is a frequent manifestation of intrinsic pathologic conditions including impairment of the blink reflex and dysfunction of lacrimal and/or oil-producing ocular glands. No matter the etiology of tear film instability, an evaporation-induced increase in the osmolarity of the preocular fluid is a universal feature of the uncomfortable sight-impairing condition of dry eye [2], which afflicts millions of individuals [3].

### 1.1. Adaptive Role of Conjunctival Goblet Cells

With intense evolutionary pressure to optimize vision, the ocular surface system possesses multiple adaptive mechanisms to respond to extracellular hyperosmolarity/dryness. Key adaptive responses include neural reflexes to increase the rate of lid blinking, the production of tears and the release of tear-stabilizing mucin. In recent studies, we focused on the adaptive role of the mucin-releasing goblet cells located in the conjunctiva of the ocular surface. When triggered by a parasympathetic reflex, these cells exocytotically release mucin-filled granules [4,5,6]. Upon entry of mucin into the microenvironment, the water-binding capacity of this glycoprotein results in formation of a muco-aqueous gel that stabilizes the preocular tear film [4].

The importance of mucin in ocular function has drawn intense scientific interest in elucidating how goblet cells transduce parasympathetic input into the exocytotic output of this tear-stabilizing glycoprotein. As a result, much is now known about the mechanism by which exocytosis is evoked by cholinergic input, which is a key mediator of the reflex triggering mucin release [5,6,7,8]. However, until recently, the role of ion channels in this neural-evoked exocytotic response remained unappreciated.

### 1.2. Impact of Ion Channels

The recent development of an experimental preparation consisting of freshly excised rat conjunctiva has facilitated study of how ion channels impact the adaptive response of goblet cells to the extracellular hyperosmolarity that is a universal feature of dry eye. These conjunctival specimens provide easy identification of goblet cells whose propensity to form tight gigaohm seals with patch-clamp micropipettes has permitted the first electrophysiological analyses of single mucin-releasing cells located within their intrinsic tissue, rather than after dissociation from neighboring cells [9,10,11]. Perforated-patch recordings of the ionic currents of conjunctival goblet cells have revealed that hyperosmotic-induced oxidants sequentially activate a series of ion channels; namely, the ATP-sensitive K^+^ (K_ATP_) channels [9], nonspecific cation (NSC) channels [9], voltage-gated calcium channels (VGCCs) [10,11] and during unremitting hyperosmolarity, P2X_7_ receptor/channels [10]. Of functional relevance, assessment of exocytotic activity via high-temporal resolution measurements of the membrane capacitance (Cm) of single goblet cells located in situ has shown that these channels modulate goblet cell function [9,10,11]. Based these new findings, as well as viability assays using conjunctival specimens [10], we recently identified four stages in the goblet cell response to levels of extracellular osmolarity associated with highly symptomatic dry eye [11].

### 1.3. Channel-Mediated Responses

During Stage 1 of the goblet cell response to hyperosmolarity, the oxidant-induced activation of hyperpolarizing K_ATP_ channels increases the membrane potential of these mucin-releasing cells from ~−37 mV to ~−52 mV [9]. In turn, this ~15-mV increase in the electro-gradient for influxing Ca^2+^ boosts the magnitude of the cholinergic-evoked exocytotic response [9], which is known to depend chiefly on the inflow of these divalent cations [6,7,12]. In Stage 2, goblet cells switch from hyperpolarized to depolarized as the progressive activation of oxidant-sensitive NSC channels lowers their membrane potential to ~−28 mV [11]. Throughout Stage 3, the goblet cell voltage remains at ~−28 mV, which is near-optimal for the tonic activation of the VGCCs [10] whose opening boosts the exocytotic response of the goblet cells to cholinergic input [10]. If normosmolarity is not restored during Stage 3, then the goblet cells enter Stage 4, which features the activation of their purinergic P2X_7_ receptor/channels [10] by ATP, which leaks from osmotically-damaged conjunctival cells [13]. Of pathobiologic importance, P2X_7_ activation markedly increases the vulnerability of the goblet cells to the lethality of hyperosmotic-induced oxidants [10]. Furthermore, the P2X_7_-induced activation of NLPR3 inflammasomes in the goblet cells [14] is a putative mechanism contributing to the production of cytokines, such as interferon-gamma, which has been found to impair production of releasable mucin [6,15,16] and over time, to compromise goblet cell viability [17,18,19]. Thus, evidence is accumulating that P2X_7_ purinoceptors play pivotal roles in the preocular inflammation and cellular death that drive progression to irreversible dry eye [2,3,16,19,20,21]. However, recent recordings of the membrane capacitance (Cm) of conjunctival goblet cells also point to P2X_7_ purinoceptors affecting the exocytotic activity of these cells [10]. Although additional data are needed, these new findings support a working model in which P2X_7_ purinoceptors exert a yin-yang impact on both the physiology and the pathobiology of the goblet cells located in the conjunctiva.

### 1.4. Research Objective

The aim of the present study was to more completely elucidate the functional impact of P2X_7_ purinoceptors on goblet cells of the conjunctiva. With neural-evoked exocytosis being the chief adaptive response of these mucin-releasing cells, this investigation focused on the exocytotic activity evoked by cholinergic input, which is the best studied parasympathetic mediator. Using high-temporal Cm measurements of single goblet cells located in freshly excised conjunctival specimens, we quantified the effect of P2X_7_ activation on the magnitude of the exocytotic response of these cells to the cholinergic agonist, carbachol. In addition, we used Cm recordings to assess the effect of P2X_7_ activation on the rate at which conjunctival goblet cells replenish their supply of releasable granules after their exocytotic depletion.

We now report that the activation of P2X_7_ purinoceptors markedly boosts cholinergic-evoked exocytosis and accelerates granule replenishment. These new observations lend support for the operational concept that by enhancing the ability of conjunctival goblet cells to supply tear-stabilizing mucin, P2X_7_ activation provides a high-gain benefit, albeit at the previously discovered risks of compromising goblet cell viability and triggering preocular inflammation.

## 2. Results

**Figure 1:**
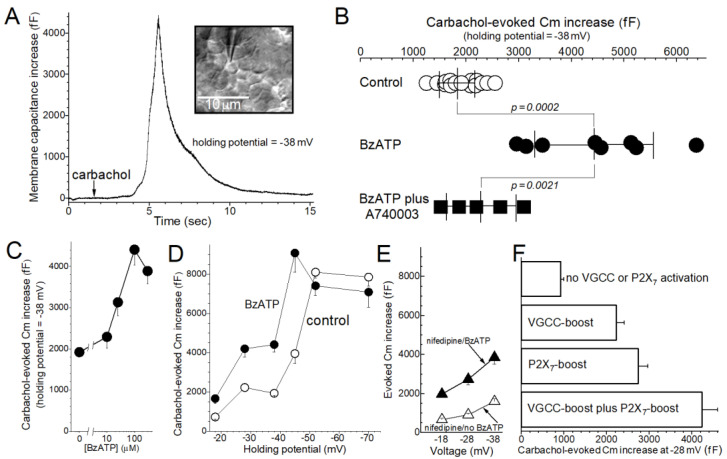
P2X_7_-induced boost in the exocytotic response of conjunctival goblet cells to cholinergic stimulation. (**A**), Example of a carbachol-evoked increase in the membrane capacitance (Cm) of a goblet cell monitored at the holding potential of −38 mV. The conjunctival specimen had been bathed for 1 h in the standard perfusate (see Section 4) supplemented with 100 µM BzATP. Arrow shows the onset of exposure to 10 µM carbachol; as previously reported this cholinergic agonist does not alter the goblet cell current [9] whose stability is required for the “sine + DC” method to accurately measure Cm [23]. Inset, differential interference contrast/infrared image of the sampled goblet cell located within the conjunctival specimen. Bar shows 10 µm. (**B**), Carbachol-evoked Cm increases monitored in conjunctival goblet cells bathed for 0.5 h to 2 h under various conditions: in the standard perfusate (control; data from Puro [9,10]), in this perfusate supplemented with 100 µM BzATP, or in the BzATP-containing perfusate supplemented with the P2X_7_ antagonist A740003 (100 nM). Horizontal lines extend ±1 SD from the mean. (**C**), Concentration of BzATP versus the carbachol-evoked Cm increase. Conjunctival specimens were bathed in a BzATP-containing solution for 30 to 80 min prior to obtaining a Cm recording. Vertical bars show SEs. *p*-values comparing the evoked Cm increase in the control solution (0 µM BzATP; *n* = 15; data from Panel B) with those measured in the presence of 10 µM (*n* = 4), 25 µM (*n* = 4), 100 µM (*n* = 8, data shown in Panel B) and 300 µM (*n* = 4) BzATP were 0.1, 0.0002, 0.0002 and <0.0001, respectively. (**D**), Effect of the goblet cell voltage on the magnitude of the carbachol-evoked Cm increase measured in the absence and in the presence of BzATP. Conjunctival specimens were maintained for 0.5 h to 2 h in the standard recording solution (control; *n* = 7 ± 2; data from Puro [9,10,11]) or in this solution supplemented with 100 µM BzATP (*n* = 5 ± 2). *p*-values comparing the control group with the BzATP group at −18 mV, −28 mV, −38 mV, −45 mV, −52 mV and −70 mV were 0.0265, 0.0010, 0.0002, 0.0034, 0.2 and 0.4, respectively. (**E**), Carbachol-evoked Cm increases in the presence of the blocker of voltage-gated calcium channels (VGCCs), nifedipine. Conjunctival specimens were maintained for 0.5 h to 2 h in the standard solution supplemented with 2 µM nifedipine without (*n* = 5 ± 1; data from Puro [10]) or with 100 µM BzATP (*n* = 4). Of note, goblet cell VGCCs are near-maximally activated at −28 mV, but minimally activated at −18 mV and −38 mV [10]. *p*-values comparing the nifedipine/no BzATP group with the nifedipine/BzATP group at −18 mV, −28 mV and −38 mV were 0.0006, 0.0031 and 0.0078, respectively. Indicative of a role for VGCCs at −28 mV, comparison of the results shown in this panel with data presented in Panel D reveals that at −28 mV, but not at −18 mV or −38 mV, nifedipine attenuated (*p* = 0.0201) the BzATP-induced boost in the carbachol-evoked Cm increase. (**F**), Carbachol-evoked Cm increases monitored at a holding potential of −28 mV, which as noted near-maximally activates goblet cell VGCCs [10]. Cm assays were performed under conditions in which neither VGCCs nor P2X_7_ purinoceptors were activated (no BzATP/+nifedipine group; *n* = 6; data from Puro [10]), only VGCCs were activated (no BzATP/no nifedipine group; *n* = 6; data from Puro [10]), only P2X_7_ purinoceptors were activated (+BzATP/+nifedipine group; *n* = 4) or both P2X_7_ purinoceptors and VGCCs were activated (+BzATP/no nifedipine group; *n* = 4). Activation of P2X_7_ purinoceptors and/or VGCCs significantly boosted (*p* ≤ 0.0056) the carbachol-evoked Cm increase. The cholinergic-evoked Cm increase measured when both VGCCs and P2X_7_ purinoceptors were activated (+BzATP/no nifedipine group) was significantly greater than the evoked Cm increases observed when only VGCCs (no BzATP/no nifedipine group; *p* = 0.0006) or only P2X_7_ purinoceptors were activated (+BzATP/+nifedipine group; *p* = 0.0138).

The aim of this study was to better understand how the activation of P2X_7_ purinoceptors affects the exocytotic response of conjunctival goblet cells to cholinergic input, which in vivo is a key parasympathetic effector triggering these cells to release of mucin-filled granules [5]. In conjunctival specimens freshly isolated from the rat, the magnitude of exocytosis evoked during exposure to the cholinergic agonist carbachol was quantified by high-temporal resolution monitoring of the membrane capacitance (Cm) of single goblet cells (Figure 1A).

Initial experiments compared the carbachol-evoked Cm increase in goblet cells located in conjunctival specimens that were bathed in a perfusate lacking or containing the P2X_7_ agonist, BzATP (100 µM). As shown in Figure 1B, BzATP significantly boosted (*p* = 0.0002) the evoked increase in Cm monitored at −38 mV, which is close to the resting membrane potential of conjunctival goblet cells under control conditions [9,10]. Indicative that this BzATP-induced boost was mediated via P2X_7_ purinoceptors, the enhancement of the carbachol-evoked exocytosis was significantly attenuated (*p* = 0.0021; Figure 1B) by the highly selective P2X_7_ antagonist A740003 (100 nM). Furthermore, similar to various P2X_7_-induced effects observed in various non-goblet cells [22], the BzATP-mediated boost in the evoked Cm increase was half-maximal at ~25 µM and maximal ~100 µM (Figure 1C).

In other experiments, we assessed how the voltage of conjunctival goblet cells affects the BzATP-induced boost in the evoked Cm increase. As summarized in Figure 1D, at holding potentials of −45 mV or less negative, BzATP (100 µM) caused significant enhancement of the evoked Cm increase. Notable is that the evoked Cm increase measured at −45 mV in the presence of the P2X_7_ agonist was ~8000 fF, which we previously reported reflects the near-total exocytotic emptying of a conjunctival goblet cell that is maximally filled with granules [9]. Hence, the magnitude of the carbachol-evoked Cm increase cannot be increased significantly beyond ~8000 fF. In addition, and also indicative that P2X_7_ activation boosts evoked exocytosis, a maximal evoked Cm increase in the absence of BzATP required a goblet cell voltage of −52 mV or more negative (Figure 1D).

Based on our earlier observation that the activation of voltage-gated calcium channels (VGCCs) boosts the cholinergic-evoked exocytotic response of conjunctival goblet cells [10], we asked whether the boosts induced by VGCCs and P2X_7_ purinoceptors are additive. VGCC activation is likely to be of relevance early in Stage 4 of the goblet cell response to hyperosmolarity since the voltage of these mucin-releasing cells is ~−28 mV [11], which is near-optimal for VGCC activation [10]. To reveal a VGCC-mediated contribution, we compared the magnitude of the BzATP-induced boost in carbachol-evoked Cm increase in the presence (Figure 1E) and absence (Figure 1D) of the VGCC blocker nifedipine (2 µM). Analysis of the results summarized in Figure 1D,E show that there is a nifedipine-sensitive component of the carbachol-evoked Cm increase measured at −28 mV, but not at −18 mV and −38 mV, which are voltages at which VGCC activity is minimal in conjunctival goblet cells [10]. Building on these findings, Figure 1F presents Cm results obtained at the holding potential of -28 mV under conditions in which neither VGCCs nor P2X_7_ receptor/channels were activated, in which either VGCCs or P2X_7_ receptor/channels were activated, or in which both VGCCs and P2X_7_ receptor/channels were activated. These findings support the conclusion that at −28 mV, the VGCC-induced boost and the P2X_7_-induced boost in carbachol-evoked exocytosis are, in fact, additive. 

**Figure 2:**
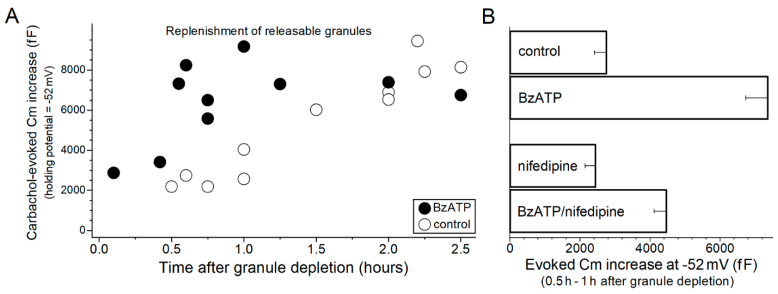
P2X_7_-induced acceleration of granule replenishment. (**A**), Time course for the replenishment of releasable granules. Conjunctival goblet cells were subjected to a granule-depleting protocol (see text) and subsequently incubated for various durations is a perfusate lacking (control) or containing 100 µM BzATP. Carbachol-evoked Cm increases were measured in goblet cells monitored at the holding potential of −52 mV, a voltage at which a near-total emptying of releasable granules is evoked by carbachol (Figure 1D and Puro [9]). (**B**), BzATP-induced acceleration of granule replenishment in the absence or in the presence of the VGCC inhibitor, nifedipine. After administration of the granule depletion protocol, conjunctival specimens were bathed for 0.5 to 1 h under various conditions: in the standard perfusate (control; *n* = 5; data from Panel A), in this perfusate supplemented with 100 µM BzATP (*n* = 5; data from Panel A), in the standard perfusate supplemented with the VGCC blocker nifedipine (*n* = 4; 2 µM) or the standard perfusate supplemented with 100 µM BzATP plus 2 µM nifedipine (*n* = 4). Indicative that a VGCC-dependent mechanism partially contributes to the BzATP-induced acceleration of granule replenishment, nifedipine diminished (*p* = 0.0068) the evoked Cm increase measured in the BzATP-treated goblet cells. Of note, in the absence of BzATP, nifedipine did not significantly affect the evoked Cm increase. Indicative that the P2X_7_-induced boost in granule replenishment also involves a VGCC-independent mechanism, BzATP induced a significant acceleration (*p* = 0.0047) in the presence of nifedipine.

High-temporal resolution measurements of goblet cell Cm also helped to establish that P2X_7_ activation impacts the rate at which releasable granules are replenished after exocytotic depletion (Figure 2). This is of importance because acceleration of granule replenishment would augment the release of tear-stabilizing mucin. To assess granule replenishment, goblet cells were initially subjected to a stimulus protocol that induced substantial depletion of stored granules. Subsequently, the amount of releasable granules was ascertained after various periods of time by measuring the carbachol-evoked Cm increase at the holding potential of −52 mV, which was chosen to optimize the magnitude of the exocytotic response (Figure 1D). More specifically, granule depletion was induced by exposing conjunctival specimens to 10 µM carbachol for 15 s at 2-min intervals for 6 min. Indicative that this protocol evokes substantial exocytosis, Cm recordings obtained at a holding potential of −38 mV, which is close to the resting membrane potential of conjunctival goblet cells in the standard perfusate [9,10,11], established that this stimulation protocol evoked a cumulative Cm increase of 5750 ± 670 fF (*n* = 4). With ~8000 fF reflecting near-total granule depletion [9], this stimulus protocol appears to empty ~70% of the releasable granules. After administration of the granule depletion protocol, conjunctival specimens were then bathed for various periods of time in the standard perfusate without or with 100 µM BzATP.

As shown in Figure 2A, under the control condition the carbachol-evoked increase in Cm took ~2 h to reach ~8000 fF, which as noted reflects near-complete replenishment of granules [9]. In contrast, granule replenishment occurred more rapidly in conjunctival specimens exposed to BzATP (Figure 2A). Namely, 0.5 h to 1 h after administration of the depletion protocol, the carbachol-evoked Cm increase was 2750 ± 340 fF (*n* = 5) in the control group (Figure 2B), but a much larger, 7360 ± 630 fF (*n* = 5; *p* < 0.0001), in the BzATP-treated group (Figure 2B).

We considered the possibility that the P2X_7_-induced acceleration of granule replenishment was, at least in part, mediated via the activation of VGCCs. A role for these depolarization-activated calcium channels was considered since P2X_7_ activation generates a depolarizing current [10] and, in addition, the activation of depolarization-activated VGCCs is known to accelerate granule replenishment [10]. Supporting a scenario in which VGCC activation plays a role, Cm recordings revealed that during a 0.5- to 2-h exposure to a perfusate containing 100 µM BzATP, the goblet cell voltage is −31 ± 1 mV (*n* = 23), which is 6 ± 1 mV depolarized (*p* < 0.0001) from their resting membrane potential in the BzATP-free solution (*n* = 17 from Puro [9,10]). Of particular interest to the present study, −31 mV is close to the optimal voltage for the activation of goblet cell VGCCs [10]. Indicative that VGCCs are involved in the P2X_7_-induced acceleration of granule replenishment, the evoked Cm increase measured 0.5 h to 1 h after granule depletion was significantly less (*p* = 0.0068) in the presence of the VGCC blocker nifedipine (Figure 2B). However, the results summarized in Figure 2B also reveal that the P2X_7_-induced acceleration of granule replenishment is not exclusively dependent upon VGCCs. Namely, even in the presence of the VGCC blocker, exposure to BzATP increased (*p* = 0.0047) the pool of releasable granules (Figure 2B). We conclude that the acceleration of granule replenishment occurring during P2X_7_ activation is mediated via VGCC-independent and VGCC-dependent mechanisms. 

Taken together, the results of this study indicate that by boosting neural-evoked exocytosis and by accelerating granule replenishment, the activation of P2X_7_ purinoceptors enhances the ability of conjunctival goblet cells to supply tear-stabilizing mucin.

## 3. Discussion

This electrophysiological study of goblet cells located in freshly excised specimens of rat conjunctiva revealed a previously unappreciated impact of P2X_7_ purinoceptors on the exocytotic function of these mucin-releasing cells. High-temporal resolution measurements of the membrane capacitance (Cm) of single goblet cells demonstrated that P2X_7_ activation markedly enhances the exocytotic response of these conjunctival cells to neural input. Cm recordings further demonstrated that the P2X_7_ agonist, BzATP, not only boosts the amount of exocytosis evoked by the cholinergic mimetic, carbachol, but also accelerates the replenishment of releasable granules after exocytotic depletion. Hence, by increasing the evoked exocytotic response and the rate of granule replenishment, the activation P2X_7_ receptor/channels facilitates the ability of conjunctival goblet cells to supply the tear-stabilizing mucin needed to restore/maintain the homeostasis of the preocular tear film. 

Discovery of the potent impact of P2X_7_ activation on neural-evoked exocytosis raises the question of when these purinoceptors become activated? Our recent study of conjunctival specimens exposed to the level of extracellular osmolarity found in individuals with symptomatic dry eye revealed that during Stage 4 of the goblet cell response to hyperosmolarity, P2X_7_ purinoceptors become activated by extracellular ATP [10], which leaks from osmotically damaged conjunctival cells [13]. Thus, this study provides evidence supporting a scenario in which P2X_7_ activation is part of the adaptive functional response of the conjunctival goblet cells to unremitting hyperosmolarity as occurs with chronic dry eye [2,3,16].

**Figure 3:**
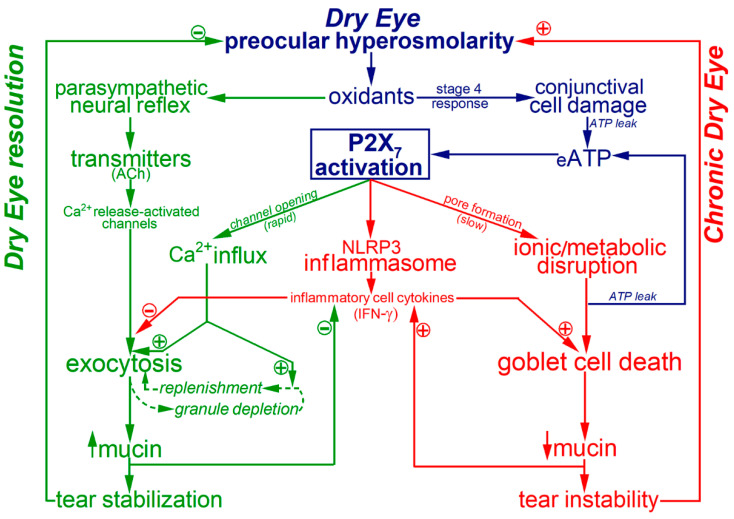
Model of the yin-yang impact of P2X_7_ purinoceptors on the adaptive and the pathobiologic responses of conjunctival goblet cells to preocular dryness/hyperosmolarity. This schematic focuses on events occurring during Stage 4 of the goblet cell response to dry eye (see Section 1). In this stage, the P2X_7_ receptor/channels of these mucin-releasing cells become activated by ATP leaking from osmotic-damaged conjunctival cells. Activated P2X_7_ receptor/channels provide pathways for the influx of Ca^2+^ whose entry is the putative mechanism by which neural-exocytosis is boosted and granule replenishment is accelerated. However, in addition to these adaptive benefits, P2X_7_ activation also exerts detrimental effects by triggering inflammation, which is important the pathogenesis of dry eye, and by compromising the viability of the goblet cells whose loss is a histopathologic characteristic of irreversible dry eye. We propose that the impact of P2X_7_ activation on the physiology and the pathobiology of conjunctival goblet cells places these key components of the ocular surface system in a ‘do-or-die’ situation. The high-gain benefit of boosting their release of tear-stabilizing mucin comes at the risk of enhancing inflammation and causing their death. In this way, we propose that P2X_7_ activation exacerbates a vicious cycle in which increasing mucin-deficiency and inflammation worsen preocular dryness/hyperosmolarity and thereby drive progression towards irreversible dry eye. Color code: shown in blue are common precursor events for the adaptive pathways (**green**) to restore tear film homeostasis as well as for the pathologic pathways (**red**) of chronic dry eye. Vertical arrows indicate changes in extracellular mucin. Stimulation and inhibition are indicated by plus and minus symbols, respectively. Abbreviations: ACh, acetylcholine; eATP, extracellular ATP; IFN-γ, interferon-gamma.

While a key conclusion of the present study is that P2X_7_ purinoceptors potently impact the physiological adaptive response of the mucin-releasing goblet cells, our previous investigations revealed that P2X_7_ activation exerts significant pathological effects. Namely, activation of these purinoceptors markedly increases the vulnerability of conjunctival goblet cells to the lethality of long-term hyperosmolarity [10], which in vivo leads to the goblet cell loss that is a histopathologic hallmark of chronic dry eye [4,19,24]. In addition, the observation by Dartt and colleagues of P2X_7_ immunoreactivity in the NLRP3 inflammasomes of rat conjunctival goblet cells [14] points to a role for these purinoceptors in the ocular surface inflammation that is an important pathogenic factor in the progression of clinical dry eye. In addition, P2X_7_-activated inflammasomes are likely to stimulate production of cytokines, such as interferon-gamma, which impairs production of releasable mucin [15] and to compromise the viability of conjunctival goblet cells [17]. Thus, while P2X_7_ activation increases the ability of goblet cells to supply the tear-stabilizing mucin for homeostasis of the preocular tear film, P2X_7_-induced goblet cell death and ocular surface inflammation promote progression to irreversible dry eye. Thus, an emerging concept is that by boosting mucin exocytosis, the activation of P2X_7_ receptor/channels in conjunctival goblet cells provides a high-gain adaptive benefit, but the activation of these purinoceptors also initiates high-risk pathobiological responses. These yin-yang effects are shown in the working model illustrated in Figure 3.

The newly appreciated impact of P2X_7_ receptor/channels on conjunctival goblet cells reveals that these purinoceptors are likely to play vital roles in the both the physiology and the pathobiology of the lacrimal functional unit. This highly interactive operational complex consists of the eye lids, lacrimal drainage system, surface epithelium, tear-secreting glands and mucin-releasing goblet cells [25]. By regulating the production, delivery and clearance of tears, the lacrimal functional unit maintains the homeostasis of the ocular surface [25]. Our experimental study indicates that a previously unsuspected mechanism involved in this homeostatic function is the P2X_7_-triggered boost in goblet cell exocytosis and granule replenishment. On the other hand, by compromising goblet cell viability and mediating inflammasome activation, P2X_7_ purinoceptors also act to impair the lacrimal functional unit. We propose that if the activation of P2X_7_ receptor/channels does not restore extracellular homeostasis relatively rapidly, then the persistent activity of these purinoceptors contributes importantly to the pathogenesis of irreversible dry eye.

Use of an experimental preparation consisting of freshly isolated conjunctival specimens has allowed us to perform the first electrophysiological analyses of single goblet cells located in their intrinsic tissue, rather than after removal from their in situ environment [9,10,11]. However, it should be noted that conclusions derived from ex vivo specimens ultimately must be confirmed in vivo, although such confirmation currently awaits technological advances. Potentially important differences between ex vivo and in vivo conditions include absence in isolated specimens of blood flow, neural reflexes and lid blinking. In addition, although we are ultimately interested in the function of human goblet cells, our quest to elucidate the roles of ion channels has begun with studies of the rat conjunctiva since appropriate human specimens are rarely available. Over the longer term, it will be necessary to establish that the adaptive and pathologic mechanisms discovered in goblet cells of the rat conjunctiva are operative in human goblet cells. Reassuringly, rat and human conjunctival goblet cells share many morphological and functional features [26,27,28] even though rat goblet cells are located in clusters and those in the human conjunctiva are physically separated [29]. A notable caveat for our experimental study is that although monitoring goblet cell Cm at the high-temporal resolution via the “sine + DC” method provides highly useful quantification of the rapid-onset exocytotic response to cholinergic stimulation, this electrophysiological technique is largely limited to the detection and analysis of the initial exocytotic response that precedes compensatory endocytosis [30]. Thus, it will be of interest in the future to develop a high-temporal resolution biochemical assay that can quantify basal and evoked mucin release over relatively long time periods in order to study sustained changes in exocytotic activity. However, it is worth noting that Cm monitoring of in situ goblet cells has opened the way for elucidating how the exocytotic activity of these vital cellular components is modulated by multiple types of ion channels including the ligand-gated P2X_7_ receptor/channels that were the focus of the present study.

The newly appreciated roles of P2X_7_ purinoceptors in the goblet cell death and the ocular inflammation that characterize irreversible dry eye raises the possibility that pharmacological targeting of these purinergic receptor/channels may provide an effective therapeutic strategy for the common distressing problem of dry eye, which both patients and clinicians agree is in need of new treatment options. However, because P2X_7_ purinoceptors not only trigger pathologic inflammation and cell death, but also markedly boost the ability of goblet cells to exocytotically supply tear-stabilizing mucin, P2X_7_ inhibition would likely impair this important adaptive response. On the other hand, a successful translational research effort to ameliorate dry eye may include pharmacological interference with the P2X_7_-driven activation of inflammasomes and the death of conjunctival goblet cells.

In summary, this electrophysiological study of rat conjunctival goblet cells has established that the activation of P2X_7_ purinoceptors enhances the magnitude of neural-evoked exocytosis and accelerates granule replenishment. While the P2X_7_-induced boost in the exocytotic capability is a high-gain benefit that helps resolve ocular dryness/hyperosmolarity, recent evidence also demonstrates that P2X_7_ activation carries high-risk due to its impact on the inflammatory processes and the goblet cell death that are closely associated with progression to irreversible dry eye. Thus, P2X_7_ activation puts goblet cells on a ‘do-or-die’ mission in which the restoration of tear film homeostasis must be achieved or else these critical cellular components of the ocular surface will be irreversibly lost. 

## 4. Materials and Methods

Experimental protocols were consistent with the Code of Practice for the Housing and Care of Animals Used in Scientific Procedures and were approval by the Institutional Animal Care and Use Committee of the University of Michigan (Protocol: PRO00008287 approved 18 April 2018). Long-Evans and Sprague–Dawley rats (Charles River, Cambridge, MA, USA) were maintained on a 12-h alternating light/dark cycle and were provided food and water ad libitum. Approximately equal numbers of males and females were used in this study.

### 4.1. Experimental Preparation

Excised specimens of rat conjunctiva were prepared as detailed [9,10]. In brief, after 3- to 6-month-old rats were euthanized by a rising concentration of carbon dioxide, two ~7-mm by ~5-mm conjunctival specimens were excised from each eye. Each specimen was promptly positioned with its exterior surface upwards in a glass-bottom recording chamber and stabilized with a harp-shaped tissue anchor (SHD26GH/10; Warner Instruments, Hamden, CT, USA). The 0.75-mL recording chambers were then filled with the standard recording solution that consisted of 105 mM NaCl, 15 mM NaHCO_3_, 10 mM Na-Hepes, 20 mM KCl, 0.5 mM CaCl_2_, 0.5 mM MgCl_2_ and 3 mM glucose; the pH was adjusted to 7.5, and with the aid of a vapor pressure osmometer (Vapro 5600, Wescor, Logan, UT, USA), osmolarity was adjusted to 300 mosM. Of note, the H^+^, K^+^, Ca^2+^ and Mg^2+^ concentrations, as well as the pH and osmolarity, reflect values measured in normal tears of many animals and humans [2,31,32,33,34]. Prior to electrophysiological recordings, specimen-containing chambers were maintained at 100% humidity and 22–23 °C in a Billups-Rothenberg modular chamber (Billups-Rothenberg, Inc., Del Mar, CA, USA).

### 4.2. Electrophysiology

As previously described [9,10,11], the exocytotic response to cholinergic stimulation was measured via perforated-patch pipettes monitoring the membrane capacitance (Cm) of single goblet cells during a 15-s exposure of a conjunctival specimen to 10 µM carbachol in our standard 300-mosM bathing solution. Since goblet cell voltage, current and exocytotic activity remain stable for prolonged periods in this normosmotic solution [9,10,11], its use facilitated detection of P2X_7_-induced effects in the absence of the potentially confounding electrophysiological responses known to be generated by conjunctival goblet cells during exposure to a hyperosmotic extracellular environment [9,10,11].

Resting membrane potentials were the zero-current voltages determined from current-voltage (I–V) plots in which the junction potential of −8 mV was adjusted off-line. More specifically, Cm and current recordings were obtained via micropipettes (TW150F-4, World Precision Instruments, Sarasota, FL, USA) that contained a solution consisting of 50 mM KCl, 65 mM K_2_SO_4_, 6 mM MgCl_2_, 10 mM K^+^-Hepes, 60 μg·mL^−1^ amphotericin B and 60 μg·mL^−1^ nystatin at pH 7.35 and 280 mosM. Under visual guidance, micropipettes formed gigaohm seals with the apical surface of cells located within a goblet cell cluster, which is a well-characterized feature of the rat conjunctiva [28,29] and is easily identified within conjunctival specimens viewed at 400× using an upright microscope equipped with differential interference contrast/infra-red optics (Figure 1A; [9]). During electrophysiological recordings, the specimen-containing chamber was perfused at ~2 mL/min with the standard perfusate solution supplemented for specific experiments with the cholinergic agonist carbachol (10 µM), the P2X_7_ agonist BzATP (10 µM to 300 µM), the P2X_7_ antagonist A740003 (100 nM), and/or the VGCC blocker nifedipine (2 µM).

I–V relations were generated by recording currents evoked by voltage steps applied via an EPC-9 patch-clamp amplifier (HEKA) controlled by Patchmaster software (HEKA Elekronik, Lambrecht, Germany). Cm was monitored by the “sine + DC” method [35] in which a 30-mV peak sinusoidal signal was applied at a frequency 1.8 kHz to a voltage-clamp recording. With Patchmaster software emulating a lock-in amplifier, the resulting current response was used to calculate Cm [36]. Patchmaster software also controlled injection of current via the recording micropipette to maintain a pre-selected holding potential throughout each Cm recording. The amplitude of the carbachol-evoked Cm increase was defined as the maximum increase in Cm observed ≤10 s after the onset of exposure to the carbachol-containing perfusate. Although a Cm change reflects net exocytosis//endocytosis, the Cm increase detected soon after exposure to the cholinergic agonist is chiefly indicative of the initially evoked exocytosis preceding endocytotic activity [30]. Cm recordings with spontaneous fluctuations of ≥100 fF were excluded. Data analysis was aided by graphics software (Origin 2017, OriginLab Corp, Northampton, MA, USA).

### 4.3. Chemicals

Chemicals were from Sigma (St. Louis, MO, USA).

### 4.4. Statistics

Probability was evaluated by Student’s two-tailed *t*-test with equal or unequal variance as appropriate.

## Data Availability

No publically archived datasets are applicable for these data.

## References

[1] Courville C.B., Smolek M.K., Klyce S.D. (2004). Contribution of the ocular surface to visual optics. Exp. Eye Res..

[2] Willcox M.D.P., Argueso P., Georgiev G.A., Holopainen J.M., Laurie G.W., Millar T.J., Papas E.B., Rolland J.P., Schmidt T.A., Stahl U. (2017). TFOS DEWS II Tear Film Report. Ocul. Surf..

[3] Clayton J.A. (2018). Dry Eye. N. Engl. J. Med..

[4] Bron A.J., De Paiva C.S., Chauhan S.K., Bonini S., Gabison E.E., Jain S., Knop E., Markoulli M., Ogawa Y., Perez V. (2017). TFOS DEWS II pathophysiology report. Ocul. Surf..

[5] Dartt D.A. (2002). Regulation of mucin and fluid secretion by conjunctival epithelial cells. Prog. Retin. Eye Res..

[6] Garcia-Posadas L., Hodges R.R., Li D., Shatos M.A., Storr-Paulsen T., Diebold Y., Dartt D.A. (2016). Interaction of IFN-gamma with cholinergic agonists to modulate rat and human goblet cell function. Mucosal. Immunol..

[7] Hodges R.R., Bair J.A., Carozza R.B., Li D., Shatos M.A., Dartt D.A. (2012). Signaling pathways used by EGF to stimulate conjunctival goblet cell secretion. Exp. Eye Res..

[8] Li D., Jiao J., Shatos M.A., Hodges R.R., Dartt D.A. (2013). Effect of VIP on intracellular [Ca^2+^], extracellular regulated kinase 1/2, and secretion in cultured rat conjunctival goblet cells. Investig. Ophthalmol. Vis. Sci..

[9] Puro D.G. (2018). Role of ion channels in the functional response of conjunctival goblet cells to dry eye. Am. J. Physiol. Cell Physiol..

[10] Puro D.G. (2020). How goblet cells respond to dry eye: Adaptive and pathological roles of voltage-gated calcium channels and P2X_7_ purinoceptors. Am. J. Physiol. Cell Physiol..

[11] Puro D.G. (2020). Bioelectric responses of conjunctival goblet cells to dry eye: Impact of ion channels on exocytotic function and viability. Int. J. Mol. Sci..

[12] Li D., Shatos M.A., Hodges R.R., Dartt D.A. (2013). Role of PKCalpha activation of Src, PI-3K/AKT, and ERK in EGF-stimulated proliferation of rat and human conjunctival goblet cells. Investig. Ophthalmol. Vis. Sci..

[13] Guzman-Aranguez A., Perez de Lara M.J., Pintor J. (2017). Hyperosmotic stress induces ATP release and changes in P2X_7_ receptor levels in human corneal and conjunctival epithelial cells. Purinergic Signal..

[14] McGilligan V.E., Gregory-Ksander M.S., Li D., Moore J.E., Hodges R.R., Gilmore M.S., Moore T.C., Dartt D.A. (2013). Staphylococcus aureus activates the NLRP3 inflammasome in human and rat conjunctival goblet cells. PLoS ONE.

[15] Coursey T.G., Tukler Henriksson J., Barbosa F.L., De Paiva C.S., Pflugfelder S.C. (2016). Interferon-gamma-induced unfolded protein response in conjunctival goblet cells as a cause of mucin deficiency in sjogren syndrome. Am. J. Pathol..

[16] Pflugfelder S.C., de Paiva C.S. (2017). The pathophysiology of dry eye disease: What we know and future directions for research. Ophthalmology.

[17] Zhang X., Chen W., De Paiva C.S., Corrales R.M., Volpe E.A., McClellan A.J., Farley W.J., Li D.Q., Pflugfelder S.C. (2011). Interferon-gamma exacerbates dry eye-induced apoptosis in conjunctiva through dual apoptotic pathways. Investig. Ophthalmol. Vis. Sci..

[18] Zhang X., De Paiva C.S., Su Z., Volpe E.A., Li D.Q., Pflugfelder S.C. (2014). Topical interferon-gamma neutralization prevents conjunctival goblet cell loss in experimental murine dry eye. Exp. Eye Res..

[19] Pflugfelder S.C., De Paiva C.S., Moore Q.L., Volpe E.A., Li D.Q., Gumus K., Zaheer M.L., Corrales R.M. (2015). Aqueous tear deficiency increases conjunctival interferon-gamma (IFN-gamma) expression and goblet cell loss. Investig. Ophthalmol. Vis. Sci..

[20] Nelson J.D., Wright J.C. (1984). Conjunctival goblet cell densities in ocular surface disease. Arch. Ophthalmol..

[21] Hessen M., Akpek E.K. (2014). Dry eye: An inflammatory ocular disease. J. Ophthalmic Vis. Res..

[22] North R.A., Surprenant A. (2000). Pharmacology of cloned P2X receptors. Annu. Rev. Pharmacol. Toxicol..

[23] Rituper B., Gucek A., Jorgacevski J., Flašker A., Kreft M., Zorec R. (2013). High-resolution membrane capacitance measurements for the study of exocytosis and endocytosis. Nat. Protoc..

[24] Moore J.E., Vasey G.T., Dartt D.A., McGilligan V.E., Atkinson S.D., Grills C., Lamey P.J., Leccisotti A., Frazer D.G., Moore T.C. (2011). Effect of tear hyperosmolarity and signs of clinical ocular surface pathology upon conjunctival goblet cell function in the human ocular surface. Investig. Ophthalmol. Vis. Sci..

[25] Pflugfelder S.C., Stern M.E. (2020). Biological functions of tear film. Exp. Eye Res..

[26] Gipson I.K. (2016). Goblet cells of the conjunctiva: A review of recent findings. Prog. Retin. Eye Res..

[27] Horikawa Y., Shatos M.A., Hodges R.R., Zoukhri D., Rios J.D., Chang E.L., Bernardino C.R., Rubin P.A., Dartt D.A. (2003). Activation of mitogen-activated protein kinase by cholinergic agonists and EGF in human compared with rat cultured conjunctival goblet cells. Investig. Ophthalmol. Vis. Sci..

[28] Setzer P.Y., Nichols B.A., Dawson C.R. (1987). Unusual structure of rat conjunctival epithelium. Light and electron microscopy. Investig. Ophthalmol. Vis. Sci..

[29] Gipson I.K., Tisdale A.S. (1997). Visualization of conjunctival goblet cell actin cytoskeleton and mucin content in tissue whole mounts. Exp. Eye Res..

[30] Smith C.B., Betz W.J. (1996). Simultaneous independent measurement of endocytosis and exocytosis. Nature.

[31] Mann A., Tighe B. (2013). Contact lens interactions with the tear film. Exp. Eye Res..

[32] Marques D.L., Alves M., Modulo C.M., Mendes da Silva L.E.C., Reinach P., Rocha E.M. (2015). Lacrimal osmolarity and ocular surface in experimental model of dry eye caused by toxicity. Rev. Bras. Oftalmol..

[33] Rismondo V., Osgood T.B., Leering P., Hattenhauer M.G., Ubels J.L., Edelhauser H.F. (1989). Electrolyte composition of lacrimal gland fluid and tears of normal and vitamin A-deficient rabbits. CLAO J..

[34] Lamkin I.D., Zimmerman K.L., Smith Fleming K.M., Martins B.C. (2020). Osmolarity of basal and reflex tears of normal dogs. Vet. Ophthalmol..

[35] Lindau M., Neher E. (1988). Patch-clamp techniques for time-resolved capacitance measurements in single cells. Pflug. Arch..

[36] Gillis K.D. (2000). Admittance-based measurement of membrane capacitance using the EPC-9 patch-clamp amplifier. Pflug. Arch..

